# Familial Hemophagocytic Lymphohistiocytosis Secondary to *PRF1* Mutation

**DOI:** 10.1155/2021/7213939

**Published:** 2021-12-29

**Authors:** Albaraa T. Alfaraidi, Abdulaziz A. Alqarni, Mohammed T. Aqeel, Turki A. Albalawi, Ahmed S. Hejazi

**Affiliations:** ^1^College of Medicine, King Saud Bin Abdulaziz University for Health Sciences, King Abdullah International Medical Research Centre, King Abdulaziz Medical City, Ministry of the National Guard-Health Affairs, Jeddah, Saudi Arabia; ^2^Department of Oncology, King Abdulaziz Medical City, Ministry of the National Guard-Health Affairs, Jeddah, Saudi Arabia

## Abstract

Hemophagocytic lymphohistiocytosis (HLH) is a life-threatening hyperinflammatory syndrome that causes systemic inflammation which can progress to multiorgan failure and death. Symptoms and signs commonly seen in HLH include high fever, hepatosplenomegaly, pancytopenia, and hypertriglyceridemia. This report describes the 8-month clinical course of a 17-year-old male with G6PD deficiency who presented with intermittent high fever of unknown origin for 8 months accompanied by pancytopenia and bilateral lower limb weakness. A pathogenic homozygous missense mutation (c.1081A > T p.(Arg361Trp)) in the *PRF1* gene was detected by whole exome sequencing (WES). The brain and the whole spine MRI showed leptomeningeal enhancement at different levels involving both the brain and the spine. Therefore, a diagnosis of familial HLH type 2 with CNS involvement was confirmed. Accordingly, treatment with dexamethasone, cyclosporin, and etoposide in addition to intrathecal methotrexate and hydrocortisone was given. The patient showed a dramatic response with significant neurological improvement of the bilateral lower limb weakness. Genetic analysis has helped the patient's family with appropriate genetic counselling. This case highlights the importance of immediate treatment with immunosuppressants and the high clinical suspicion of physicians regarding HLH in areas where consanguinity is common.

## 1. Introduction

Hemophagocytic lymphohistiocytosis (HLH) is a life-threatening hyperinflammatory syndrome characterized by hypercytokinemia and lymphohistiocytic proliferation. In patients with HLH, dysregulated hyper-reactive immune cells cause systemic inflammation that can progress to multiorgan failure and death. The altered interaction between natural killer (NK) cells, CD 8+ cytotoxic T cells, and their antigens leads to a vicious cycle of inflammation which recruits more cytotoxic cells. This results in hypercytokinemia that causes widespread activation of macrophages and hemophagocytosis [1–4]. With mortality rates between 42% and 88%, due to the nature of the disease, prompt initiation of immunosuppressants is essential as soon as a diagnosis is confirmed [5]. HLH is common in infancy; however, it has been reported among all age groups [6]. The inflammatory syndrome has been grouped into two types: familial and acquired HLH. Familial HLH is an autosomal recessive condition in which consanguineous marriage plays an important role in inheriting the disease-causing mutation. It occurs due to a mutation in the genes responsible for normal natural killer (NK) cell activity and the cytotoxic function of T lymphocytes. The acquired form of HLH has been associated with infectious, autoinflammatory, autoimmune, and metabolic diseases [7]. Patients with HLH may present with nonspecific signs of inflammation such as fever, malaise, and fatigue. HLH is characterized by edema, hepatosplenomegaly, and liver dysfunction. Laboratory studies may show pancytopenia, coagulation abnormalities, hypofibrinogenemia, and hypertriglyceridemia [2]. We report this case of a 17-year-old male diagnosed with familial HLH due to a mutation in the *PRF1* gene with a literature review of the current treatment options and an emphasis on the importance of genetic counselling in families affected by HLH.

## 2. Case Presentation

**Figure 1:**
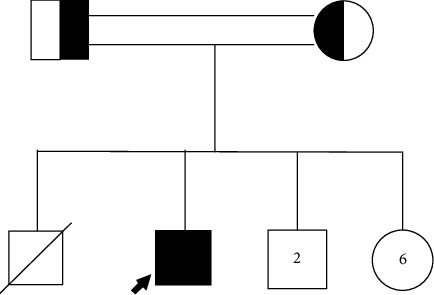
Pedigree chart of the proband.

A 17-year-old male, known to have glucose-6-phosphate dehydrogenase (G6PD) deficiency, was admitted to our hospital due to pancytopenia accompanied by a high-grade intermittent fever of unknown origin for the last eight months which was not relieved by antipyretics. The patient had significant weight loss accompanied by generalized weakness and myalgia. On physical examination, the patient looked sick, pale, and cachectic. Abdominal examination was remarkable for hepatosplenomegaly. On neurological examination, the patient had bilateral lower limb weakness with a power of 4 out of 5 in both limbs. Blood laboratory studies showed pancytopenia, hypertriglyceridemia (3.13 mmol/L), and hyperferritinemia (1912 µg/L). Other laboratory studies showed increased liver enzymes, low fibrinogen (1.4), and increased cerebrospinal fluid (CSF) total protein concentration (1.37, reference range: 0.15–0.45 g/L) (Table 1). In our patient, the soluble interleukin-2 receptor (sIL-2R) level was significantly elevated (15900 U/mL). The viral workup which included CMV and hepatitis A, B, and C serological markers was negative. A bone marrow biopsy was performed which revealed a hypercellular bone marrow with prominent hemophagocytic activity and an increased number of histiocytic cells. The proband is an offspring of first-cousin parents with no previously documented genetic disease in the family (Figure 1). The brain and the whole spine MRI showed leptomeningeal enhancement at different levels involving both the brain and the spine.

The patient was assumed to be a case of HLH with CNS involvement as he met the diagnostic criteria of HLH-2004. Accordingly, treatment with dexamethasone, cyclosporin, and etoposide in addition to intrathecal methotrexate and hydrocortisone was started. The first two doses of etoposide treatment were reduced to 50% due to high bilirubin levels (Table 1). The patient showed a dramatic response after starting treatment and had an uneventful treatment course. Symptomatically, the patient became afebrile and vitally stable. On day 20 of treatment, ferritin levels improved to 881 *μ*g/L, and hemoglobin, platelets, WBC, and LFTs had improved (Table 1). After completion of the first two months of treatment, the patient showed a significant neurological improvement in terms of his bilateral lower limb weakness. Postinduction MRI of the brain and the whole spine showed a partial improvement of the previously seen leptomeningeal enhancement.

To determine whether our patient is a case of familial HLH, genomic DNA was extracted from peripheral blood and was sent for whole exome sequencing (WES). WES identified that our patient had a homozygous missense mutation (NM_001083116.3 (PRF1): c.1081A > T, p.Arg361Trp) in the *PRF1* gene, which is classified as pathogenic and has been previously described in the literature in a family with HLH (PMIDs: 14757862, 23592409, 32542393, 27577878, and 15755897). Considering the homozygous pathogenic variant in *PRF1* and the supportive phenotype of the patient, a genetic diagnosis of familial HLH type 2 was confirmed (OMIM: 603553). Parental samples for which WES was equally performed revealed that parents were heterozygous for the identified mutation. Therefore, an autosomal recessive pattern of inheritance was confirmed in the family. Given the confirmed diagnosis of familial HLH disease with CNS involvement, the patient was a candidate for an allogeneic bone marrow transplant and HLA typing, and a search for a compatible stem cell donor was carried out. Screening of all siblings by WES revealed an 8-year-old HLA-matched sister who had a negative WES for the relevant mutations. Accordingly, the patient was offered an allogeneic bone marrow transplant.

## 3. Discussion

Familial HLH are a group of autosomal recessive diseases that occur frequently in consanguineous families [8]. Pathological variants in *PRF1*, *UNC13D*, *STX1*, and *STXBP2* have been linked to cause different forms of familial HLH (familial HLH2-HLH5). Familial HLH type 2 is the most common in this group and accounts for 20–40% of all familial HLH cases [9]. These forms of familial HLH lead to a defect in lymphocyte granule-mediated cytotoxicity [10]. Perforin gene mutations were the first genetic causes of familial HLH which were discovered in 1999 [11]. About half of the primary HLH cases were attributed to *PRF1* mutations [2, 12]. *PRF1* variants have been described in the literature in other diseases such as multiple sclerosis, non-Hodgkin's lymphoma, and leukemia [13–15]. The reported incidence of *PRF1* mutations that cause FHLH-2 varies between different ethnic groups [12].

In our patient, whole exome sequencing (WES) found that our patient had a homozygous missense mutation (NM_001083116.3 (PRF1): c.1081 A > T, p.Arg361Trp) in the *PRF1* gene. The *PRF1* gene is located on chromosome 10q22.1 and codes for the perforin protein which is responsible for lymphocyte granule-mediated cytotoxicity [10]. In patients with perforin deficiency, the immune system is unable to kill the target cells as the released contents of the cytotoxic granules cannot penetrate these cells. Mutations in *UNC13D*, *STX11*, and *STXBP2* genes cause familial HLH types 3 to 5, respectively, and lead to defective lymphocyte granule-mediated cytotoxicity [10].

According to the revised diagnostic criteria of the HLH-2004 protocol, HLH is assumed if a genetic defect consistent with HLH has been found or clinical and laboratory criteria are met. Etoposide, steroids, and cyclosporin A with or without intrathecal methotrexate are considered first-line therapy for patients with HLH [16]. CNS involvement in HLH has been associated with poorer prognosis and long-term neurological sequelae [17]. The frequency of CNS involvement varies widely in the literature and ranges from 10% to 73% of patients [17, 18]. Histopathological changes may range from minimal infiltration of leptomeninges by macrophages and lymphocytes to advanced infiltration of the parenchyma and tissue necrosis. The most important finding is the presence of hemophagocytosis, which is frequently observed in leptomeninges [19].

Currently, the only long-term curative treatment for primary HLH is allogeneic bone marrow transplantation. The HLH steering committee of the Histiocyte Society urgently recommends consulting a stem cell transplant expert about the treatment options offered since these patients carry a high risk of reactivation even after the acute episode has subsided [20].

## 4. Conclusion

The diagnosis of HLH remains challenging as patients present with nonspecific symptoms. Without a bone marrow transplant, patients with HLH have a lifelong risk of relapse. The lack of a gold standard confirmatory test for HLH makes it difficult to diagnose HLH in an emergency setting. High clinical suspicion of physicians is essential in areas where consanguinity is common if a patient presents with a fever of unknown origin accompanied by clinical features and laboratory studies that are suggestive of HLH. Immediate treatment with immunosuppressants can be lifesaving and should be provided once a diagnosis has been reached. If indicated, a prenatal analysis may be offered to the family of the patient. Testing for HLH mutations in siblings and family members should be performed before being considered as donors. Genetic counselling regarding future children is recommended to be offered to consanguineous parents with a family history of individuals affected by familial HLH.

## Figures and Tables

**Table 1:** An overview of relevant laboratory parameters during the clinical course of the patient.

Parameters	Reference range	Admission Day 0	Day 5	Day 1 of HLH-04 protocol	Day 5 of HLH-04 protocol	Day 10 of HLH-04 protocol	Day 15 of HLH-04 protocol	Day 20 of HLH-04 protocol	Day of discharge
Hemoglobin (g/-dL)	13.0–18.0	8.7	7.8	10.8	10.2	9.7	8.2	8.5	9.5
Neutrophils (10^9^xL)	2.0–7.5	0.8	0.99	0.75	0.92	1.02	0.79	0.42	3.44
Lymphocytes (10^9^/L)	1.5–4.0	1.61	1.07	1.44	2.3	0.48	0.41	0.84	2.16
Platelet count (10^9^/L)	150–450	63	54	41	87	68	69	221	273
Ferritin (*μ*g/L)	11–172	1420	1912	948	1821	1612	976	881	371
Fibrinogen (g/L)	2.0–4.0	—	1.5	1.6	0.9	1.2	1.4	1.6	1.9
Triglyceride (mmol/L)	0.5–2.20	3.13	—	—	—	—	—	—	—
Direct bilirubin (*μ*mol/L)	1.7–6.7	20	80	—	—	—	—	—	34
ALT (IU/L)	9–24	502	707	611	323	303	257	274	128
AST (IU/L)	14–35	354	608	390	189	113	79	89	63
ALP (IU/L)	59–164	994	885	967	631	413	305	309	253
GGT (IU/L)	7–21	250	219	569	424	535	647	782	1025
sIL-2r (U/ml)	158–623	—	—	—	15900	—	—	—	—

## Data Availability

Any inquiries about the availability of data presented in this study may be directed to the corresponding author.

## Abbreviations

HLH: Hemophagocytic lymphohistiocytosis
PRF1: Perforin 1
FHL: Familial hemophagocytic lymphohistiocytosis
WES: Whole exome sequencing
G6PD: Glucose-6-phosphate dehydrogenase
ALT: Alanine transaminase
AST: Aspartate transaminase
ALP: Alkaline phosphatase
GGT: Gamma-glutamyl transferase
PT: Prothrombin time
TG: Triglycerides
LDH: Lactate dehydrogenase
RBCs: Red blood cells
CMV: Cytomegalovirus
MRI: Magnetic resonance imaging
PIDs: Primary immunodeficiencies
IUIS: International Union of Immunological Societies
sIL-2r: Soluble interleukin-2 receptor
HLA: Human leukocyte antigen
HSCT: Hematopoietic stem cell transplantation.
